# Demographic and psychosocial correlates of measurement error and reactivity bias in a 4-d image-based mobile food record among adults with overweight and obesity

**DOI:** 10.1017/S0007114522001532

**Published:** 2023-02-28

**Authors:** Clare Whitton, Janelle D. Healy, Satvinder S. Dhaliwal, Charlene Shoneye, Amelia J. Harray, Barbara A. Mullan, Joanne A. McVeigh, Carol J. Boushey, Deborah A. Kerr

**Affiliations:** 1 Curtin School of Population Health, Curtin University, Kent Street, GPO Box U1987, Perth 6845, Australia; 2 Curtin Health Innovation Research Institute, Curtin University, Kent Street, GPO Box U1987, Perth 6845, Australia; 3 Singapore University of Social Sciences, 463 Clementi Road, 599494, Singapore; 4 Institute for Research in Molecular Medicine (INFORMM), Universiti Sains Malaysia, 11800 Minden, Pulau Pinang, Malaysia; 5 Duke-NUS Medical School, National University of Singapore, 8 College Rd, Singapore 169857, Singapore; 6 Telethon Kids Institute, 15 Hospital Ave, Nedlands, WA 6009, Australia; 7 Enable Institute, Curtin University, Kent Street, GPO Box U1987, Perth 6845, Australia; 8 Curtin School of Allied Health, Curtin University, Kent Street, GPO Box U1987, Perth 6845, Australia; 9 Movement Physiology Laboratory, University of Witwatersrand, Johannesburg, South Africa; 10 Epidemiology Program, University of Hawaii Cancer Center, Honolulu, HI, USA

**Keywords:** Food record, Misreporting, Image-based dietary assessment, Misestimation, Low energy reporting

## Abstract

Improving dietary reporting among people living with obesity is challenging as many factors influence reporting accuracy. Reactive Reporting may occur in response to dietary recording, but little is known about how image-based methods influence this process. Using a 4-d image-based mobile food record (mFR^TM^), this study aimed to identify demographic and psychosocial correlates of measurement error and reactivity bias, among adults with BMI 25–40 kg/m^2^. Participants (*n* 155, aged 18–65 years) completed psychosocial questionnaires and kept a 4-d mFR^TM^. Energy expenditure (EE) was estimated using ≥ 4 d of hip-worn accelerometer data, and energy intake (EI) was measured using mFR^TM^. EI:EE ratios were calculated, and participants in the highest tertile were considered to have Plausible Intakes. Negative changes in EI according to regression slopes indicated Reactive Reporting. Mean EI was 72 % (sd = 21) of estimated EE. Among participants with Plausible Intakes, mean EI was 96 % (sd = 13) of estimated EE. Higher BMI (OR 0·81, 95 % CI 0·72, 0·92) and greater need for social approval (OR 0·31, 95 % CI 0·10, 0·96) were associated with lower likelihood of Plausible Intakes. Estimated EI decreased by 3 % per d of recording (interquartile range − 14 %,6 %) among all participants. The EI of Reactive Reporters (*n* 52) decreased by 17 %/d (interquartile range − 23 %,–13 %). A history of weight loss (> 10 kg) (OR 3·4, 95 % CI 1·5, 7·8) and higher percentage of daily energy from protein (OR 1·1, 95 % CI 1·0, 1·2) were associated with greater odds of Reactive Reporting. Identification of reactivity to measurement, as well as Plausible Intakes, is recommended in community-dwelling studies to highlight and address sources of bias.

Errors in measurement and estimation of usual dietary intakes have been observed in self-reported dietary assessment methods^(1–3)^. Measurement error in dietary assessment results in inaccurate estimates of food, energy and nutrient intakes, compromising the reliability of dietary surveillance data, diet–health relationships observed in epidemiology and nutrition intervention evaluations. Despite being widely acknowledged, there are gaps in understanding the drivers of measurement error, which has prevented the development of mitigation strategies.

Measurement error, particularly misestimation of energy intake (EI), has been observed in people with obesity, more so than among people with a lower BMI^(4)^. In attempts to explain the relationship between BMI and misestimation of EI, many psychological and psychosocial factors have been investigated. For example, associations have been reported between misestimation of EI and high cognitive restraint^(5,6)^, social desirability^(7)^, fear of negative evaluation^(8)^, poor body image^(9)^, perceived stress^(10)^, and depression^(11)^. The reasons for the association between BMI and misestimation of EI remain unclear, and further studies within populations living with overweight and obesity are needed.

Most dietary interventions conducted with community populations use self-report measures such as food records, due to considerations regarding participant burden and cost. However, the recording process is known to result in reactivity bias, a change in behaviour in response to being observed^(12)^, also referred to as the ‘observation effect’. Another component is misreporting. Identifying misreporting of EI in community-dwelling studies, without the use of controlled conditions or biomarkers, has frequently been achieved using estimates of energy expenditure (EE), such as accelerometer data or energy requirement equations based on basal metabolic rate and body mass^(13,14)^. These methods have enabled research into the determinants of misreporting, such as personal, demographic, social desirability and psychological characteristics. However, methods to identify reactivity bias in community populations have rarely been developed and undertaken, and there is no such body of research on determinants of reactivity bias.

Recently, reactivity to measurement was described as a neglected source of bias in trials, and it was recommended that risk of reactivity be identified, and followed up with quantitative investigation if necessary^(15)^. However, standardised methods to identify reactivity bias in both dietary interventions and large-scale studies of diet are lacking. To date, reactivity bias has been detected and quantified in a few dietary studies, using a range of techniques. In residential feeding studies, EI was 5–6 % lower when participants were overtly observed, compared with when they were covertly observed^(16,17)^, but these studies did not report whether the effect of observation changed over time. In a metabolic study among women, reported EI was 16 % lower than EE, and this was solely attributed to reactivity bias based on changes in body mass and accuracy of water intake reporting^(18)^. This study did not report the effect of study day on misestimation of EI, so it is unclear whether the magnitude increased over time in response to recording. Findings from two US studies in community populations suggested that reactivity bias may increase in magnitude over a period of observation. Rebro *et al.* (1998) reported that in 4-d non-consecutive food records, US women recorded significantly fewer snacks and food items/ingredients overall on the last day of a dietary record as compared with the first day^(19)^. In contrast, Kirkpatrick *et al.* (2012) observed no reactivity bias in a 4-d food record, but slight declines in the number of food items reported over time in 7-d and 30-d food checklists. However, this study did not consider other possible sources of measurement error^(20)^.

In previous dietary studies investigating reactivity bias, study participants were asked to keep written food records. Digital technologies have enabled participants to capture images of their food and beverage consumption, and such methods are frequently used in dietary studies^(21)^. The mobile food record (mFR^TM^) is an image-based dietary assessment method where participants capture before and after eating images of eating occasions^(22–24)^. A community-dwelling study designed to test the accuracy of the mFR^TM^ with EE using the doubly labelled water method, demonstrated the mFR^TM^ was comparable to other studies with written dietary records. A unique aspect of the mFR^TM^ method is that the portion size estimation from the images is undertaken either by automated methods or a human-trained analyst, rather than the participant as is the case in the present study^(22)^. How this image review process may influence energy misestimation in participants living with obesity is unclear. Furthermore, it is unclear whether reactivity bias occurs with the mFR^TM^, to what extent, and whether it increases with the length of the recording period.

The present study of adults living with overweight and obesity aimed to: (1) identify patterns of dietary intake measurement accuracy in a 4-d image-based mFR^TM^, including reactivity to measurement; and (2) determine demographic and psychosocial correlates of measurement accuracy.

## Methods

### Participants and study design

We used baseline data from 160 participants enrolled in the ‘Tailored Diet and Activity study’ (ToDAy), a 1-year diet and physical activity randomised controlled trial in Perth, Western Australia. The protocol is described in detail elsewhere^(25)^. Briefly, participants were recruited via social media, letterbox drops and radio interviews. Eligible participants were aged 18–65 years, had a BMI of 25–40 kg/m^2^ and owned a smartphone with Internet access. Participants with serious illnesses or medical conditions or weight loss > 4 kg in the previous 2 months were ineligible. Participants using appetite suppressants, weight loss or hormone replacement medication were also ineligible. Baseline data were collected before randomisation into the intervention and active control groups. During the first study visit, anthropometric measures were collected, and participants received training on using the mFR^TM^ application and a hip-worn accelerometer. ToDAy was registered with the Australian New Zealand Clinical Trials Registry (ACTRN12617000554369). All study protocols were approved by the Curtin University Human Research Ethics Committee (approval number HR61/2016).

### Demographic and psychosocial measures

Prior to the first study visit, participants were invited to complete online demographic, lifestyle and psychosocial questionnaires. Data were collected on gender identity, age, highest level of educational attainment, ethnicity, socio-economic status, income and smoking status.

#### Three-Factor Eating Questionnaire

Assessment of eating behaviour (cognitive restraint, hunger and disinhibition) was conducted using the fifty-one-item Three-Factor Eating Questionnaire^(26)^. Cognitive restraint refers to the conscious restriction of dietary intake, hunger refers to the desire to eat and disinhibition refers to a loss of self-control. Good reliability of Three-Factor Eating Questionnaire subscales (Cronbach’s *α* > 0·7) has been demonstrated in populations with obesity^(27,28)^.

#### Depression, Anxiety, and Stress Scales (DASS-21)

Participants also completed the Depression, Anxiety, and Stress Scales (DASS-21)^(29)^, a twenty-one-item scale used to measure current states of depression, anxiety and stress. Each subscale has high reliability and high convergent validity with other measures of depression and anxiety^(30)^.

#### Weight loss history questionnaire

Participants completed an eight-item weight loss history questionnaire^(31)^ assessing the frequency, nature and short-term success of previous weight loss attempts.

#### Social Desirability Scale

A short thirteen-item version^(32)^ of the Social Desirability Scale^(33)^ was completed, to measure the need for social approval and acceptance. The shortened scale shows internal consistency (Kuder–Richardson Formula 20 coefficient = 0·76) and is highly correlated (*r* = 0·93, *P* < 0·001) with the original thirty-three-item scale^(32)^. Higher scores indicate a tendency to provide responses in questionnaires or interviews that are socially acceptable rather than objective and that present an individual in a favourable light^(34)^.

#### Fear of Negative Evaluation Scale

Participants completed a Fear of Negative Evaluation Scale^(35)^, a twelve-item scale assessing the level of concern a person has about others’ opinions of them. Higher scores indicate a greater level of concern about being negatively evaluated. The Fear of Negative Evaluation Scale has demonstrated a high level of internal consistency (Cronbach’s *α* = 0·90) and a test–retest reliability coefficient of 0·75 over a 4-week interval^(35)^.

### Dietary assessment

At the first study visit, participants received training and were instructed to record their dietary intake over four consecutive days using the mFR^TM^, an image-based food record application^(24)^. Participants were asked to capture ‘before eating’ and ‘after eating’ images of all foods and beverages consumed over four consecutive days, including at least one weekend day. As in other studies^(36,37)^, Friday was considered a weekend day because Friday alcohol intake resembles weekend alcohol intake^(38)^. Participants were instructed that images were to contain a fiducial marker (an object of known shape, size and colour)^(24)^, to aid in portion size estimation. Approximately 1 week later, during the second study visit, participants returned and a dietitian (CS, DAK and JDH) clarified the contents of the images where they were unclear and probed for any food and beverages not captured or unclear/hidden. A trained analyst dietitian (JDH) selected matching food codes and estimated portion sizes based on the contents of the images and the review with participants. In a validation study of EI from the mFR using doubly labelled water, estimated EI was significantly correlated with total EE (*r* = 0·58, *P* < 0·0001)^(22)^. Nutrition analysis software (FoodWorks 9, Xyris Software) which was linked to the Australian Food Composition Database, AUSNUT 2011–13, was used. Mean daily intakes of energy and nutrients were calculated.

### Other measures

During the first study visit, participants were instructed to wear a triaxial accelerometer (GT3X+, Actigraph) on their right hip for seven consecutive days without removal during sleeping. The Actigraph GT3X+ is a reliable, research-grade tool for measuring physical activity in community-dwelling conditions^(39)^. The GT3X+ was programmed to record raw data at a frequency of 30 Hz. Data were later reduced to vertical axis movement counts per 60 s epoch for the current analysis. Participants who provided at least 4 d with at least 10 h of wear time per d were included in the analyses. Cut-off points were used to classify each minute of accelerometer data as sedentary (< 100 counts per minute)^(40)^, light intensity (100–1951 counts per minute), moderate intensity (1952–5724 counts per minute) or vigorous intensity (> 5724 counts per minute), and total metabolic equivalent of task (MET) minutes were calculated using the Freedson equation^(13)^. One MET is equivalent to uptake of approximately 3·5 ml oxygen per kg body weight per minute, with consumption of 1 l of oxygen being equivalent to approximately 5 kcal^(41)^. Thus, each participants’ mean daily MET minutes were multiplied by their body weight (kg) and oxygen uptake (3·5 ml), and divided by 200, to calculate estimated average daily EE in kilocalories. Weight and height were measured according to established protocols^(42)^ during the second study visit.

### Statistical analysis

For each participant, the ratio of estimated mean daily EI and EE was calculated. Intakes of carbohydrate, protein, total fat, saturated fat and alcohol as a percentage of daily EI were calculated by multiplying grams by 37 kJ for fats, 19 kJ for protein and carbohydrate, and 29 kJ for alcohol, then dividing by total kJ × 100. Tertiles for EI:EE ratio were calculated, and participants in the tertile with the highest EI:EE ratio were considered to have Plausible Intakes. In order to examine reactivity to recording, linear regression models of changes in EI over time were constructed for each participant, regressing EI (kJ) against recording days (1, 2, 3 and 4). Resulting unstandardised β-coefficients indicated the gradient of change in EI per unit time (day) and were used to categorise participants into tertiles. The lowest tertile which contained negative β-coefficients were considered to be Reactive Reporters.

Univariate logistic regression was conducted to assess associations of all demographic and psychosocial characteristics with the odds of having Plausible Intakes and with the odds of being a Reactive Reporter. It was calculated that with a total of 152 participants, there would be at least 80 % power (at a 5 % level of significance) of detecting a correlation of at least 0·25 between continuous variables, and a difference in proportions of at least 25 % for categorical variables. Characteristics with a *P*-value < 0·25 were considered for inclusion in the multivariate regression model. In the multivariate model, a backward regression procedure was used with a cut-off of 0·1 on the likelihood ratio test. This cut-off was used to prevent the loss of potentially important variables, which may have resulted if a more stringent cut-off was used. Subsequently, variables with the highest P-values in the final step of the backwards model were removed one at a time to establish the impact on other variables. Interactions between variables in the final step were considered, and interaction terms assessed where necessary. All statistical analyses were conducted in IBM SPSS Statistics 26 (IBM Corp).

## Results

A total of 155 participants provided at least 3 d of dietary data and at least 4 d of valid accelerometer data. Participants were female (68 %), highly educated (60 % held a bachelor’s degree or higher) and had never smoked cigarettes (70 %) (Table 1). The mean daily EI was 7280 kJ, sd 2008 kJ (1740 kcal, sd 480 kcal) and the mean BMI was 31·2 kg/m^2^ (sd 4·0 kg/m^2^). Among participants whose food records covered both weekdays and weekend days (*n* 139), there was no difference between EI on weekend days (7632 kJ (equivalent to 1824 kcal)) as compared with weekdays (7452 kJ (equivalent to 1781 kcal), *P* = 0·5).

**Table 1.** Characteristics of ToDAy participants at baseline (Numbers and percentages, *n* 155)

Characteristic	*n*	%
Gender		
Male	49	32
Female	106	68
Age group (years)		
19–29	12	8
30–59	118	76
≥ 60	25	16
Highest level of educational attainment		
Year 12 or lower	27	17
Trade or diploma	35	23
Bachelor’s degree or higher	93	60
Total annual household income (AUD$)		
$0–$99 999	52	34
$100 000 or more	93	60
Prefer not to answer	10	6
Smoking status		
Never smoked regularly	108	70
Current or former smoker	47	30
BMI category		
25–29·99 (kg/m^2^)	71	46
≥ 30 kg/m^2^	84	54
Number of days of food record		
3 d	10	6
4 d	145	94

### Plausible Intakes

The mean EI:EE ratio was 0·72 (sd = 0·21). Participant characteristics across tertiles of EI:EE ratio were generally similar (Table 2), apart from differences in BMI, and percentage energy from protein. In the lowest tertile (EI:EE ratio ≤ 0·64), BMI was at least 2 BMI points higher (*P* = 0·001) compared with the other tertiles. Percentage energy from protein was approximately 2 percentage points higher in the lowest EI:EE ratio tertile (*P* = 0·004) as compared with the other tertiles. The total amount of time between non-consecutive record days was slightly higher by approximately half a day in the lowest tertile of EI:EE ratio, as compared with in the other tertiles (*P* = 0·025).

**Table 2.** Demographic, lifestyle and psychosocial characteristics of ToDAy participants at baseline, by tertiles of EI:EE ratio (Numbers and percentages, *n* 155)

	Tertiles of EI:EE ratio						
	Tertile 1 (lowest) EI:EE ≤ 0·64, *n* 52		Tertile 2 EI:EE 0·65–0·79, *n* 52		Tertile 3 (highest) EI:EE ≥ 0·80 ‘Plausible Intakes’, *n* 51		
Characteristic	*n*	%	*n*	%	*n*	%	*P* *
Demographics							
Age (years)							0·89
Mean	47·0		47·9		47·8		
se	1·5		1·5		1·7		
Gender, *n* (%)							0·52
Male	18	34·6	18	34·6	13	25·5	
Female	34	65·4	34	65·4	38	74·5	
Highest level of educational attainment, *n* (%)							0·38
Year 12 or lower	10	19·2	8	15·4	9	17·6	
Trade or diploma	16	30·8	11	21·2	8	15·7	
Bachelor’s degree or higher	26	50·0	33	63·5	34	66·7	
Total annual household income, n (%)							0·58
AUD$0–$99 999	13	25·0	21	40·4	18	35·3	
AUD$100 000 or more	35	67·3	28	53·8	30	58·8	
Prefer not to answer	4	7·7	3	5·8	3	5·9	
Smoking status, *n* (%)							0·69
Never smoked regularly	34	65·4	38	73·1	36	70·6	
Current or former smoker	18	34·6	14	26·9	15	29·4	

	Mean	se	Mean	se	Mean	se	
BMI (kg/m^2^)	32·9	0·6	30·6	0·5	30·0	0·5	0·001
Accelerometry							
Accelerometer wear time (days)	7·2	0·3	7·6	0·3	7·1	0·2	0·34
Moderate–vigorous activity time (% of accel. wear time)	3·5	0·3	3·5	0·3	2·9	0·2	0·13
Sedentary time (% of accel. wear time)	66·7	1·0	65·4	0·9	64·5	1·0	0·28
Accelerometer wear time (min/d)	945·9	11·3	923·4	8·4	915·5	11·3	0·10
Estimated daily energy expenditure, derived from accelerometer (kcal)	2732·0	78·7	2427·2	59·8	2246·3	50·4	< 0·001
Mobile food record							
Average daily energy intake, derived from food record (kcal)	1347·8	48·2	1735·2	44·3	2147·1	54·9	< 0·001
Energy from protein (%)	20·6	0·6	18·7	0·5	18·2	0·5	0·004
Energy from total fat (%)	36·3	0·8	35·9	0·8	35·9	0·8	0·94
Energy from saturated fat (%)	14·1	0·4	13·7	0·4	13·2	0·3	0·32
Energy from carbohydrates (%)	37·0	1·2	39·0	0·9	39·4	1·0	0·21
Energy from alcohol (%)	3·0	0·6	2·7	0·5	3·2	0·5	0·81
	*n*	%	*n*	%	*n*	%	
Psychosocial measures							
Previous weight loss, *n* (%)							0·62
≤ 10 kg	30	57·7	31	59·6	34	66·7	
More than 10 kg	22	42·3	21	40·4	17	33·3	
Social desirability score, tertile							0·27
Tertile 1 (lowest)	15	28·8	9	17·3	19	37·3	
Tertile 2	12	23·1	13	25·0	10	19·6	
Tertile 3 (highest)	25	48·1	30	57·7	22	43·1	
Fear of negative evaluation score, tertile							0·12
Tertile 1 (lowest)	16	30·8	14	26·9	19	37·3	
Tertile 2	16	30·8	23	44·2	10	19·6	
Tertile 3 (highest)	20	38·5	15	28·8	22	43·1	
Depression score, category							0·97
Normal	32	61·5	33	63·5	31	60·8	
Mild	10	19·2	7	13·5	10	19·6	
Moderate	6	11·5	6	11·5	4	7·8	
Severe	3	5·8	4	7·7	3	5·9	
Extremely severe	1	1·9	2	3·8	3	5·9	
Anxiety score, category							0·09†
Normal	46	88·5	41	78·8	44	86·3	
Mild	1	1·9	6	11·5	1	2·0	
Moderate	5	9·6	2	3·8	5	9·8	
Severe	0	0·0	3	5·8	0	0·0	
Extremely severe	0	0·0	0	0·0	1	2·0	
Stress score, category							0·68
Normal	45	86·5	42	80·8	39	76·5	
Mild	4	7·7	5	9·6	5	9·8	
Moderate	1	1·9	4	7·7	5	9·8	
Severe	2	3·8	1	1·9	1	2·0	
Extremely severe	0	0·0	0	0·0	1	2·0	
Hunger score, category							0·53
Low	33	63·5	37	71·2	37	72·5	
High	9	17·3	9	17·3	10	19·6	
Clinical	10	19·2	6	11·5	4	7·8	
Restraint score, category							0·25
Low	33	63·5	37	71·2	40	78·4	
High	19	36·5	15	28·8	11	21·6	
Disinhibition score, category							0·81
Low	17	32·7	20	38·5	19	37·3	
High	35	67·3	32	61·5	32	62·7	
Operational factors							
Food record duration (days)							0·48
3	5	9·6	2	3·8	3	5·9	
4	47	90·4	50	96·2	48	94·1	
Weekend days included in food record (days)							
Mean	1·29		1·60		1·61		0·10
se	0·12		0·12		0·12		
Day type of food record day 1							0·81
Weekday	31	59·6	28	53·8	30	58·8	
Weekend	21	40·4	24	46·2	21	41·2	
Day type of final day of food record							0·19
Weekday	33	63·5	32	61·5	24	47·1	
Weekend	19	36·5	20	38·5	27	52·9	
Total time between non-consecutive record days (days)							
Mean	1·02		0·58		0·67		0·03
se	0·2		0·18		0·23		

EI, energy intake; EE, energy expenditure.

* *P*-values are derived from chi-squared tests for categorical variables, from ANOVA for normally distributed continuous variables and from Kruskal–Wallis tests for non-normally distributed continuous variables.

† Chi-square test excluded ‘severe’ and ‘extremely severe’ categories due to low numbers.

In multivariate analyses, higher BMI (OR 0·81, 95 % CI 0·72, 0·92), greater social desirability scores (OR 0·31, 95 % CI 0·10, 0·96) and moderate *v*. low fear of negative evaluation by others (OR 0·17, 95 % CI 0·06, 0·54) were all associated with lower likelihood of having Plausible Intakes (EI:EE ratio tertile ≥ 0·80) (Table 3). No associations were detected between other participant characteristics and the likelihood of having Plausible Intakes.

**Table 3.** Associations between participant characteristics and Plausible Intakes among ToDAy participants at baseline (Odd ratio and 95 % confidence intervals, *n* 155)

	Univariate†			Multivariable analyses‡,§			Final model§,||			
Independent variable*	OR	95 % CI	*P*	OR	95 % CI	*P*	OR	95 % CI		*P*
		Lower, upper			Lower, upper			Lower, Upper		
BMI (kg/m^2^)	0·88	0·80, 0·97	0·01	0·83	0·72, 0·95	0·007	0·81	0·72, 0·92		0·001
Energy from protein (%)	0·89	0·81, 0·99	0·03	0·88	0·77, 1·00	0·05				
Energy from saturated fat (%)	0·92	0·82, 1·04	0·19	0·89	0·75, 1·05	0·17				
Social desirability score, tertile (Ref: tertile 1, lowest)			0·19			0·06				0·12
Social desirability, tertile 2	0·51	0·20, 1·31	0·16	0·52	0·15, 1·77	0·30	0·60	0·20, 1·82		0·37
Social desirability, tertile 3 (highest)	0·51	0·23, 1·10	0·09	0·22	0·06, 0·78	0·02	0·31	0·10, 0·96		0·04
Fear of negative evaluation score, tertile (Ref: tertile 1, lowest)			0·09			0·01				0·008
Fear of negative evaluation, tertile 2	0·41	0·16, 1·00	0·05	0·15	0·04, 0·53	0·004	0·17	0·05, 0·54		0·003
Fear of negative evaluation, tertile 3 (highest)	0·99	0·45, 2·17	0·99	0·51	0·16, 1·68	0·27	0·56	0·20, 1·59		0·27
Anxiety score, category (Ref: normal)¶			0·78			0·89				
Anxiety score, mild	0·28	0·03, 2·37	0·24	0·32	0·03, 3·68	0·36				
Anxiety score, moderate	1·41	0·42, 4·71	0·57	1·41	0·21, 9·50	0·72				
Anxiety score, severe	0·00	0·00	1·00	0·00	0·00	1·00				
Stress score, category (Ref: normal)¶			0·80			0·74				
Stress score, mild	1·24	0·39, 3·94	0·72	0·82	0·14, 4·76	0·83				
Stress score, moderate	2·23	0·61, 8·15	0·23	2·09	0·32, 13·47	0·44				
Stress score, severe	0·74	0·08, 7·38	0·80	0·46	0·02, 9·95	0·62				
Restraint score, category (high *v*. low)	0·57	0·26, 1·24	0·15	0·80	0·28, 2·27	0·67				
Hunger score, category (Ref: low)			0·43			0·22				
Hunger score, high *v*. low	1·05	0·44, 2·51	0·91	0·67	0·21, 2·15	0·50				
Hunger score, clinically high *v*. low	0·47	0·15, 1·52	0·21	0·28	0·06, 1·22	0·09				

Ref, reference category.

* Dependent variable is being in highest tertile (≥ 0·80) of energy intake:energy expenditure ratio, *v*. being in tertile 1 or 2.

† For the following variables *P* > 0·25 in univariate analyses, so they were not included in multivariable models: energy from total fat, %; energy from carbohydrates, %; energy from alcohol, %; highest level of educational attainment; total annual household income; smoking status; depression score category; disinhibition score category and previous weight loss.

‡ First step of backwards regression model included BMI (continuous), energy from protein (continuous), energy from saturated fat (continuous), social desirability score (categorical), fear of negative evaluation score (categorical), anxiety score (categorical), stress score (categorical), restraint score (categorical) and hunger score (categorical).

§ Both multivariable models were adjusted for the covariates: age; gender; accelerometer wear time, min/d; food record duration, days; weekend days included in food record, days; total time between non-consecutive record days, days; day type of food record day 1 (weekend *v*. weekday); day type of final day of food record (weekend *v*. weekday).

|| Final step of backwards regression model included BMI (continuous), social desirability score (categorical) and fear of negative evaluation score (categorical).

¶ 'Extremely severe categories for anxiety and stress are not shown because of low numbers.

### Reactive Reporting

On average, EI decreased by 3 % per d (interquartile range (IQR) − 14 %, 6 %). Participants in the lowest tertile of EI change (ranging from −3910 to −762 kJ per d) (online Supplementary Fig. 1) were classified as Reactive Reporters, with a median change in EI equivalent to a decrease of 17 % per d (IQR − 23 %, −13 %). Tertile 2 ranged from −721 to 271 kJ per d, and tertile 3 ranged from 272 to 2280 kJ per d. Characteristics of participants according to tertile of change in EI are shown in Table 4. Mean daily EI of Reactive Reporters were significantly lower (mean difference 267 kcal, *P* = 0·01) than intakes in the opposite tertile. A significantly larger proportion of Reactive Reporters had a history of substantial weight loss (> 10 kg), with more than 25 percentage points difference, compared with the other two tertiles (*P* = 0·002). There was also a significantly larger proportion of Reactive Reporters (81 %) with low *v*. high disinhibition scores compared with in the opposite tertile (57 %, *P* = 0·021).

**Table 4.** Demographic, lifestyle and psychosocial characteristics of ToDAy study participants at baseline, by tertiles of change in energy intake over the recording period (Numbers and percentages, *n* 155)

	Tertiles of change in energy intake						
	Tertile 1		Tertile 2		Tertile 3		
	–3910 to −762 kJ/d ‘Reactive Reporters’ *n* 52		–721 to 271 kJ/d *n* 52		272 to 2280 kJ/d *n* 51		
Characteristic	*n*	%	*n*	%	*n*	%	*P* *
Demographics							
Age (years)							
Mean	46·1		46·8		49·8		0·18
se	1·5		1·5		1·6		
Gender, *n* (%)							0·87
Male	15	28·8	17	32·7	17	33·3	
Female	37	71·2	35	67·3	34	66·7	
Highest level of educational attainment, *n* (%)							0·66
Year 12 or lower	10	19·2	6	11·5	11	21·6	
Trade or diploma	12	23·1	11	21·2	12	23·5	
Bachelor’s degree or higher	30	57·7	35	67·3	28	54·9	
Total annual household income, *n* (%)							0·10
$0–$99 999	18	34·6	13	25·0	21	41·2	
$100 000 or more	30	57·7	33	63·5	30	58·8	
Prefer not to answer	4	7·7	6	11·5	0	0·0	
Smoking status, *n* (%)							0·24
Never smoked regularly	38	73·1	39	75·0	31	60·8	
Current or former smoker	14	26·9	13	25·0	20	39·2	

	Mean	se	Mean	se	Mean	se	
BMI (kg/m^2^)	32·0	0·6	30·1	0·4	31·3	0·6	0·21
Accelerometry							
Accelerometer wear time (days)	7·1	0·2	7·1	0·2	7·7	0·3	0·16
Moderate-vigorous activity time (% of accel wear time)	3·2	0·3	3·4	0·3	3·3	0·3	0·73
Sedentary time (% of accel wear time)	66·1	0·9	65·4	0·9	65·1	1·0	0·78
Accelerometer wear time (min/d)	925·6	11·8	921·3	9·1	938·4	10·5	0·49
Estimated daily energy expenditure, derived from accelerometer (kcal)	2555·4	73·7	2382·5	62·7	2472·0	71·4	0·21
Mobile food record							
Average daily energy intake, derived from food record (kcal)	1631·9	66·5	1694·9	60·8	1898·4	68·6	0·01
Energy from protein (%)	20·0	0·5	19·1	0·7	18·4	0·5	0·10
Energy from total fat (%)	36·7	0·8	35·3	0·8	36·1	0·9	0·45
Energy from saturated fat (%)	14·1	0·4	13·4	0·4	13·6	0·4	0·43
Energy from carbohydrates (%)	37·7	1·1	39·3	1·1	38·3	1·0	0·53
Energy from alcohol (%)	2·4	0·4	2·9	0·6	3·5	0·6	0·35

	*n*	%	*n*	%	*n*	%	
Psychosocial measures							
Previous weight loss, *n* (%)							0·002
≤ 10 kg	22	42·3	36	69·2	37	72·5	
More than 10 kg	30	57·7	16	30·8	14	27·5	
Social desirability score, tertile							0·53
Tertile 1 (lowest)	18	34·6	13	25·0	12	23·5	
Tertile 2	13	25·0	10	19·2	12	23·5	
Tertile 3 (highest)	21	40·4	29	55·8	27	52·9	
Fear of negative evaluation score, tertile							0·34
Tertile 1 (lowest)	12	23·1	17	32·7	20	39·2	
Tertile 2	21	40·4	16	30·8	12	23·5	
Tertile 3 (highest)	19	36·5	19	36·5	19	37·3	
Depression score, category							0·13
Normal	29	55·8	37	71·2	30	58·8	
Mild	14	26·9	3	5·8	10	19·6	
Moderate	6	11·5	5	9·6	5	9·8	
Severe	2	3·8	3	5·8	5	9·8	
Extremely severe	1	1·9	4	7·7	1	2·0	
Anxiety score, category							0·56
Normal	44	84·6	40	76·9	47	92·2	
Mild	3	5·8	3	5·8	2	3·9	
Moderate	4	7·7	6	11·5	2	3·9	
Severe	1	1·9	2	3·8	0	0·0	
Extremely severe	0	0·0	1	1·9	0	0·0	
Stress score, category							0·14
Normal	41	78·8	44	84·6	41	80·4	
Mild	8	15·4	1	1·9	5	9·8	
Moderate	2	3·8	3	5·8	5	9·8	
Severe	1	1·9	3	5·8	0	0·0	
Extremely severe	0	0·0	1	1·9	0	0·0	
Hunger score, category							0·97
Low	36	69·2	37	71·2	34	66·7	
High	10	19·2	8	15·4	10	19·6	
Clinical	6	11·5	7	13·5	7	13·7	
Restraint score, category							0·20
Low	18	34·6	27	51·9	21	41·2	
High	34	65·4	25	48·1	30	58·8	
Disinhibition score, category							0·02
Low	10	19·2	13	25·0	22	43·1	
High	42	80·8	39	75·0	29	56·9	
Operational factors							
Food record duration (days)							0·48
3	5	9·6	2	3·8	3	5·9	
4	47	90·4	50	96·2	48	94·1	
Weekend days included in food record (days)	1·54	0·12	1·5	0·13	1·45	0·11	0·92
Day type of food record day 1							0·26
Weekday	28	53·8	27	51·9	34	66·7	
Weekend	24	46·2	25	48·1	17	33·3	
Day type of final day of food record							0·44
Weekday	30	57·7	33	63·5	26	51·0	
Weekend	22	42·3	19	36·5	25	49·0	
Total time between non-consecutive record days (days)							
Mean	0·81		0·35		1·12		0·14
se	0·18		0·09		0·29		

* *P*-values are derived from chi-squared tests for categorical variables, from ANOVA for normally distributed continuous variables and from Kruskal–Wallis tests for non-normally distributed continuous variables.

A history of substantial weight loss (> 10 kg) (OR 3·4, 95 % CI 1·5, 7·8), mild *v*. no depression (OR 4·2, 95 % CI 1·5, 12·1) and moderate *v*. low fear of negative evaluation by others (OR 3·8, 95 % CI 1·4, 10·4) were associated with increased likelihood of being a Reactive Reporter (Table 5). Participants with a higher percentage of EI from protein were more likely to be Reactive Reporters (OR 1·1, 95 % CI 1·0, 1·2). No other associations were detected between participant characteristics and the likelihood of Reactive Reporting. The interaction between BMI and weight loss history was not associated with Reactive Reporting. Most participants with Plausible Intakes (78 %) were not classified as Reactive Reporters. More than one-quarter (26 %) of participants had Plausible Intakes with no evidence of Reactive Reporting.

**Table 5.** Associations between participant characteristics and Reactive Reporting among ToDAy study participants at baseline (Odd ratio and 95 % confidence intervals, *n* 155)

	Univariate†			Multivariable analyses‡,§			Final model§,||		
Independent variable*	OR	95 % CI	*P*	OR	95 % CI	*P*	OR	95 % CI	*P*
		Lower, upper			Lower, upper			Lower, upper	
BMI (kg/m^2^)	1·08	1·00, 1·18	0·06	1·02	0·91, 1·14	0·72			
Energy from protein (%)	1·09	1·00, 1·19	0·06	1·11	1·00, 1·24	0·05	1·11	1·00, 1·22	0·04
Energy from saturated fat (%)	1·08	0·96, 1·22	0·22	1·02	0·87, 1·20	0·78			
Energy from alcohol (%)	0·94	0·85, 1·04	0·22	0·95	0·83, 1·09	0·49			
Previous weight loss, category (> 10 kg *v*. ≤ 10 kg)	3·32	1·66, 6·65	0·001	3·55	1·33, 9·51	0·01	3·44	1·52, 7·79	0·003
Social desirability score, tertile (Ref: tertile 1, lowest)			0·24			0·83			
Social desirability, tertile 2	0·82	0·33, 2·05	0·67	0·87	0·26, 2·87	0·81			
Social desirability, tertile 3 (highest)	0·52	0·24, 1·14	0·10	0·70	0·21, 2·29	0·56			
Fear of negative evaluation score, tertile (Ref: tertile 1, lowest)			0·16			0·04			0·04
Fear of negative evaluation, tertile 2	2·31	0·98, 5·48	0·06	4·27	1·37, 13·28	0·01	3·75	1·36, 10·38	0·01
Fear of negative evaluation, tertile 3 (highest)	1·54	0·66, 3·62	0·32	1·81	0·54, 6·09	0·34	1·72	0·60, 4·92	0·32
Depression score, category (Ref: normal)			0·20			0·08			0·03
Depression score, mild	2·49	1·04, 5·95	0·04	4·17	1·29, 13·50	0·02	4·24	1·49, 12·10	0·007
Depression score, moderate	1·39	0·46, 4·17	0·56	2·04	0·51, 8·20	0·32	1·63	0·46, 5·77	0·45
Depression score, severe	0·58	0·12, 2·89	0·50	0·36	0·04, 3·11	0·36	0·37	0·05, 2·54	0·31
Depression score, extremely severe	0·46	0·05, 4·13	0·49	0·38	0·02, 6·28	0·50	0·38	0·03, 4·30	0·43
Stress score, category (Ref: normal)¶			0·38			0·21			
Stress score, mild	2·76	0·90, 8·49	0·08	3·22	0·68, 15·25	0·14			
Stress score, moderate	0·52	0·11, 2·55	0·42	0·20	0·03, 1·46	0·11			
Stress score, severe	0·69	0·07, 6·85	0·75	1·29	0·07, 24·16	0·87			
Restraint score, category (high *v*. low)	1·65	0·83, 3·29	0·16	1·46	0·58, 3·68	0·42			
Disinhibition score, category (high *v*. low)	2·16	0·97, 4·82	0·06	1·89	0·64, 5·62	0·25			

Ref, reference category.

* Dependent variable is being in first tertile (T1) of change in energy intake over recording period, *v*. being in tertile 2 or 3.

† For the following variables *P* > 0·25 in univariate analyses, so they were not included in multivariable models: energy from total fat, %; energy from carbohydrates, %; highest level of educational attainment; total annual household income; smoking status; anxiety score category and hunger score category.

‡ First step of backwards regression model included BMI (continuous), energy from protein (continuous), energy from saturated fat (continuous), energy from alcohol (continuous), previous weight loss (categorical), social desirability score (categorical), fear of negative evaluation score (categorical), depression score (categorical), stress score (categorical), restraint score (categorical) and disinhibition score (categorical).

§ Both multivariable models were adjusted for the covariates: age; gender; accelerometer wear time, min/d; dood record duration, days; weekend days included in food record, days; total time between non-consecutive record days, days; day type of food record day 1 (weekend *v*. weekday); day type of final day of food record (weekend *v*. weekday).

|| Final step of backwards regression model included energy from protein (continuous), previous weight loss (categorical), fear of negative evaluation score (categorical) and depression score (categorical).

¶ Extremely severe categories for stress are not shown because of low numbers.

## Discussion

The aim of this study was to identify patterns of dietary intake measurement error in a community-dwelling setting and identify demographic and psychosocial factors associated with these patterns in adults living with overweight and obesity. Higher BMI and the need for social approval were inversely associated with having Plausible Intakes. We observed variation in the extent of reactivity to recording dietary intake with the mFR and found that a history of losing more than 10 kg body weight was associated with Reactive Reporting. In contrast, BMI was not associated with Reactive Reporting.

The magnitude of the dietary intake measurement error that we observed was comparable to other studies of populations with overweight and obesity. For example, in the current study, EI was 72 % of EE, while in a doubly labelled water study evaluating a technology-based dietary record in Australian women with overweight and obesity, EI was 80 % of expenditure^(43)^. This suggests that the use of accelerometer-derived EE was a valid method to evaluate the accuracy of EI from self-reported dietary intake. The average extent of Reactive Reporting in the current study was similar to observations of Kirkpatrick *et al.* (2012) using a 7-d food checklist, in which the reported frequency of consumption of total food items declined slightly over the recording period (–2 % per d)^(20)^. Unlike that study, Reactive Reporting in the current study was identified within a 4-d study period using the mFR and was shown to increase over the study period.

Higher BMI was associated with a lower likelihood of having Plausible Intakes, a finding observed in many previous studies^(44–46)^. Our findings demonstrate that among people with overweight and obesity, there continues to be an inverse association between BMI and the accurate estimation of dietary intakes. However, our findings also show that among populations with overweight and obesity, there are participants whose estimated dietary intakes are plausible and without reactivity bias (26 % of participants in the present study). Among populations with overweight and obesity, associations between weight status and under-estimation of EI were observed in Australian women aged 70–80 years who kept a 3-d weighed food record^(47)^ but not among Canadian women aged 46–69 years who kept a 3-d estimated food record^(10)^. In the Canadian study, fat mass and perceived stress were associated with lower likelihood of accurate estimation of dietary intake. In a US study, women aged 22–42 years with obesity kept a 7-d estimated food record, and results indicated that depression but not BMI predicted misestimation of EI^(11)^. Reasons for these discrepancies in associations between BMI and dietary intake measurement error are unclear but suggest the presence of other underlying factors. For example, it has been suggested that the association between weight status and accurate estimation of dietary intake is underpinned by awareness of the types and amounts of foods consumed^(8)^.

In the present study, there was no association between BMI and Reactive Reporting. Similarly, other studies have found little evidence of a role of BMI in Reactive Reporting^(19,20)^. However, previous weight loss attempts of more than 10 kg were associated with greater likelihood of Reactive Reporting, compared with having never lost 10 kg. History of weight loss attempts and frequent weight fluctuations have been associated with total error in the measurement of dietary intake^(45)^. To our knowledge, the current study is the first to examine and detect an association between weight loss history and reactivity bias. Dietary self-monitoring is known to bring about dietary behaviour change through raising awareness of intake and is a key behaviour change technique in weight management^(48,49)^. In a recent systematic review of randomised controlled trials, fostering awareness and attention during eating, known as mindful eating, were associated with weight loss^(50)^. Thus, it is conceivable that participants with experience of dietary self-monitoring and a history of weight loss, when asked to undertake dietary recording in the current study, reacted by changing their dietary intake.

A higher percentage of energy derived from protein was associated with higher likelihood of Reactive Reporting. It was also associated with lower likelihood of Plausible Intakes in univariate analysis but was not retained in the final multivariable model, possibly due to low power. Previous research has found that protein intake is misestimated to less extent than EI^(51)^. As such, higher contributions of protein to total EI among underreporters has been observed, as compared with among participants with plausible intakes^(10,52–54)^. This may be because high protein foods are often part of a main meal, and using mFR, participants have reported remembering to take images of snacks as being more difficult than meals^(55)^. On the other hand, Reactive Reporting may be characterised by reduced overall intake of carbohydrate and fat-rich food choices. A US study found that women recorded significantly fewer snacks on the last day of a dietary record as compared with the first day^(19)^. Further investigation is required into the patterns of reporting of foods, meals and snacks over time, to better understand the sources of bias that exist.

Our study used an image-based mFR^TM^, in which foods and beverages present in each image were assessed for descriptions and portion sizes by a trained analyst. As such, participants had a reduced role in the process of dietary reporting as compared with traditional food record or 24-h recall methods in which all description and quantification of foods/beverages originate from participants. Misestimation errors may have occurred through several sources. Participants may have reacted to the reporting process by either reducing their intake or forgetting to take images of consumed items. In usability research, participants have reported remembering to take images of snacks as being more difficult than meals^(55)^. Our finding on protein may indicate that participants continued to take images of main meals but were more likely to omit images of smaller eating occasions, and this requires further investigation. Misestimating EI may have occurred from inaccurate description or quantification of items in images. Previous research has shown foods of amorphous shape and higher density are more difficult to estimate from images^(56)^. Future improvements in automated methods to estimate EI may improve the accuracy of image-based dietary assessment methods^(57)^.

The Social Desirability Scale assessed the tendency to provide responses in questionnaires or interviews that are socially acceptable rather than objective, and that present an individual in a favourable light^(34)^. In our study, higher social desirability scores were associated with lower likelihood of having Plausible Intakes, which aligns with numerous other studies on social desirability and reporting accuracy, across dietary assessment methodologies and across population subgroups^(7,8,51,58–60)^. In contrast, we found no association between social desirability scores and Reactive Reporting. This indicates that factors underlying Reactive Reporting and having Plausible Intakes may not be synonymous, and that the presence and prediction of Reactive Reporting in dietary data require separate attention in order to improve data quality and reliability.

We found that a moderate score on the Fear of Negative Evaluation Scale was associated with higher odds of Reactive Reporting and lower odds of having Plausible Intakes. This finding is difficult to interpret, as we would expect to see a trend including the highest scores. Similarly, in a recent study in a group of weight-stable participants with a range of BMI, psychological factors (personality, social desirability, body image, intelligence quotient and eating behaviour) were weakly and inconsistently associated with measurement error when diet was assessed using a range of methods (weighed food record, 24-h recall, FFQ and diet history)^(17)^. The results on the depression score in the current study in relation to Reactive Reporting may also be spurious due to low variation in depression scores and the small number of participants with higher scores. As such, we believe that the results of the current study on depression and fear of negative evaluation score do not warrant interpretation.

This study had several strengths and limitations to be considered when interpreting the findings. We considered total measurement error and reactivity bias in the same sample using the same demographic and psychosocial measurements. This allowed us to demonstrate that there are some distinct characteristics associated with reactivity bias as opposed to total measurement error. The method we used for categorising individuals based on the extent of Reactive Reporting of dietary intakes (online Supplementary Fig. 1) provides a novel contribution to the literature and aligns with recent recommendations on exploring reactions to measurement in trials^(15)^. Our study sample of people with overweight and obesity was highly suitable for the assessment of dietary intake measurement error, reactivity bias and psychosocial correlates, given that previous research observed high frequency of measurement error in such populations. Nevertheless, the self-selected sample in our study may not represent the wider population. For example, a greater proportion of our participants were educated to the bachelor’s degree level or higher than in the general population (60 % *v*. 24 %)^(61)^. Our study was powered to detect a difference in proportions of at least 25 % for categorical variables. As such, we may not have detected smaller effects and recommend that future studies are conducted with larger sample sizes to increase the power. Our study used baseline data from participants enrolled in a weight loss intervention, and although recent weight loss was an exclusion criteria, study enrolment alone may have induced some change in usual behaviour. For example, it is possible that some participants began restricting their EI before commencing with the mFR. However, if the extent of restriction increased in response to keeping the 4-d mFR, then this behaviour is also considered to be reactivity to measurement, the outcome measure we were attempting to capture. Furthermore, increased physical activity levels have been observed in control groups of weight loss interventions^(62)^, possibly because study enrolment and/or study measurements provide some motivation to increase activity levels. Some evidence suggests that accelerometer wear causes an increase in activity levels^(63,64)^, and this may have occurred in our study. This could have resulted in misclassification of some participants in the lower two tertiles of EI:EE ratio. However, studies that detected an increase in activity as a response to accelerometer wear reported that moderate to vigorous physical activity was less affected than light activity and sedentary time^(65,66)^. This suggests the impact on estimation of average daily EE in our study is likely to be minor.

In conclusion, BMI and the need for social approval were inversely associated with plausible estimates of EI using an image-based mFR. Image-based technology reduces energy misestimation, but challenges in collecting reliable self-reported dietary data remain. A change in behaviour in response to being observed (Reactive Reporting) is a distinct type of dietary intake measurement error. Variation in Reactive Reporting between individuals presents a challenge in addressing this bias at the group level. Our study adds to the literature by demonstrating a practical method, for categorising individuals based on the extent of Reactive Reporting displayed in multiple days of dietary data. This method may be applied in future large-scale dietary studies and surveys, to enable better understanding and management of measurement error.

## Supplementary material

For supplementary material accompanying this paper visit https://doi.org/10.1017/S0007114522001532. click here to view supplementary material
