# Supplementary material for: Demographic and psychosocial correlates of measurement error and reactivity bias in a 4-d image-based mobile food record among adults with overweight and obesity
Source: Br J Nutr. 2022 May 19;129(4):725–36. doi: 10.1017/S0007114522001532 (PMC9899562; doi:10.1017/S0007114522001532)
Supplement: Supplementary file 1 [file S0007114522001532sup.zip › S0007114522001532sup001.docx]

**Supplementary Material**

Supplementary Figure 1. Energy intake (kcal/day) of each participant (n = 155) by study day, in each tertile of change in energy intake over time. (A) Tertile 1; (B) Tertile 2; (C) Tertile 3. Each line represents an individual participant. Participants in Tertile 1 were categorised as Reactive Reporters.

| 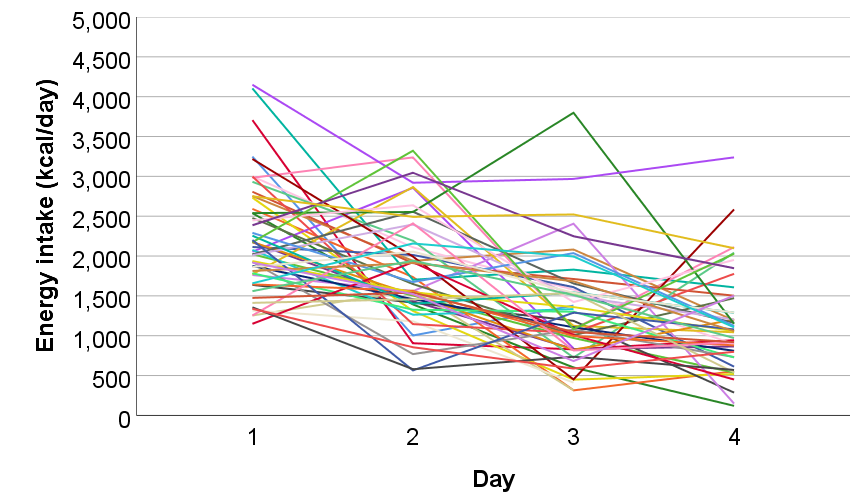 | 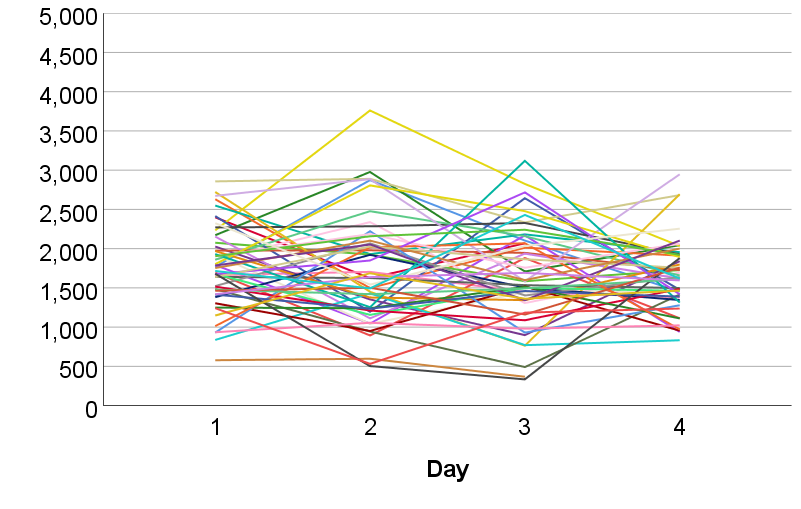 | 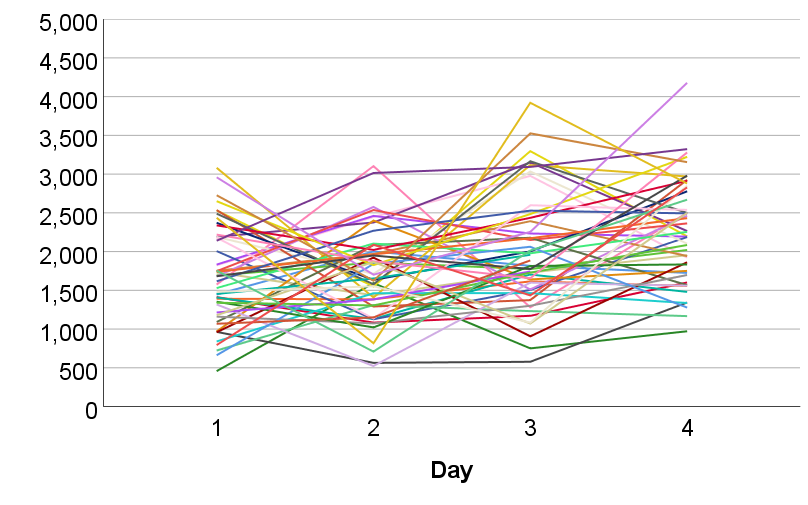 |
| --- | --- | --- |
| (A) | (B) | (C) |
